# Human subarachnoid space width oscillations in the resting state

**DOI:** 10.1038/s41598-018-21038-0

**Published:** 2018-02-15

**Authors:** Marcin Gruszecki, Gemma Lancaster, Aneta Stefanovska, J. Patrick Neary, Ryan T. Dech, Wojciech Guminski, Andrzej F. Frydrychowski, Jacek Kot, Pawel J. Winklewski

**Affiliations:** 1 Department of Radiology Informatics and Statistics, Medical University of Gdansk, Gdansk, Poland; 2 Department of Physics, Lancaster University, Lancaster, UK; 3 Faculty of Kinesiology and Health Studies, University of Regina, Regina, Canada; 4 Department of Computer Communications, Faculty of Electronics, Telecommunications and Informatics, Gdansk University of Technology, Gdansk, Poland; 5 Department of Human Physiology, Medical University of Gdansk, Gdansk, Poland; 6 National Centre for Hyperbaric Medicine, Institute of Maritime and Tropical Medicine, Medical University of Gdansk, Gdynia, Poland; 7 Department of Clinical Anatomy and Physiology, Pomeranian University of Slupsk, Slupsk, Poland

## Abstract

Abnormal cerebrospinal fluid (CSF) pulsatility has been implicated in patients suffering from various diseases, including multiple sclerosis and hypertension. CSF pulsatility results in subarachnoid space (SAS) width changes, which can be measured with near-infrared transillumination backscattering sounding (NIR-T/BSS). The aim of this study was to combine NIR-T/BSS and wavelet analysis methods to characterise the dynamics of the SAS width within a wide range of frequencies from 0.005 to 2 Hz, with low frequencies studied in detail for the first time. From recordings in the resting state, we also demonstrate the relationships between SAS width in both hemispheres of the brain, and investigate how the SAS width dynamics is related to the blood pressure (BP). These investigations also revealed influences of age and SAS correlation on the dynamics of SAS width and its similarity with the BP. Combination of NIR-T/BSS and time-frequency analysis may open up new frontiers in the understanding and diagnosis of various neurodegenerative and ageing related diseases to improve diagnostic procedures and patient prognosis.

## Introduction

Below the scalp and the skull lie the dura mater, the arachnoid, and the pia mater. The space between the arachnoid and the pia mater is known as the subarachnoid space (SAS) and is filled with translucent cerebrospinal fluid (CSF). CSF plays a very important role in the prevention of injury, acting as a cushion and circulating within the ventricular system of the brain. It has been shown in a number of radiological studies that CSF circulation is affected by heart and respiration driven changes in the cardiovascular system^1–8^. This can be explained by considering the cerebral circulation in more detail. Blood vessels that serve the brain must first go through the SAS and cross the pia mater before penetrating the brain^9^. Therefore, from a mechanical perspective, any changes in the volume of these blood vessels must result in CSF motion. In turn, CSF motion leads directly to SAS width fluctuations. For example, during the cardiac systolic phase, blood volume in the cerebral vessels increases. As the brain is enclosed in a rigid skull, any increase in blood volume needs to be accompanied by a displacement of an approximately equal amount of CSF into the compliant spinal compartment to prevent an increase in intracranial pressure^1,3^. This induces oscillations in the CSF with the same frequency as the heartbeat. Breathing-driven CSF oscillations, including changes in SAS width, have also been described in a number of studies^2,4,5^.

Previous studies revealed six different frequency intervals corresponding to different physiological oscillations of the vessels. Intervals I (0.6–2 Hz) and II (0.145–0.6 Hz) are related to cardiac and respiratory function, respectively. Interval III (0.052–0.145 Hz) is usually associated with smooth muscle cell activity while interval IV (0.021–0.052 Hz) has been proposed to reflect smooth muscle autonomic innervation^10^. There is one structural component in common throughout the entire cardiovascular system, a smooth inner vessel lining of endothelial cells. The endothelium is located at the interface between the blood and the vessel wall. Furchgott and Zawadzki^11^ showed that intact endothelium produces a factor that causes relaxation of vascular smooth muscle. This factor is nitric oxide (NO) and is released continuously by the endothelium in the arterioles and arteries, contributing to vasodilation in the basal state. The production of NO can be stimulated by acetylcholine or by mechanical effects, such as increase of blood flow or pressure. Endothelial effects manifest in intervals V (nitric oxide (NO) dependent) and VI (NO independent) (0.0095–0.021 and 0.005–0.0095 Hz, respectively)^12,13^. Considering that the SAS width is directly affected by the volume of cerebral vessels, it is likely that signatures of these blood flow oscillations will also be transmitted to the CSF, and thus be observable as SAS width changes.

CSF oscillations were first characterised by Lundberg^6^. Lundberg *et al*. described three types of waves: A, B and C^14,15^. A waves, or plateau waves, are usually associated with transient decline in neurological function. B waves occur periodically with a frequency of 0.25–0.33 Hz and are associated with cardiorespiratory dynamics^8,16^. C waves are entirely synchronous with blood pressure Traube-Hering-Meyer waves (0.1 Hz) and are seen in patients with severe intracranial hypertension or brain stem dysfunction. The presence of C waves is associated with loss of autoregulation^7^. Due to technological limitations and short recordings, earlier studies did not assess CSF oscillations below the frequency of Lundberg’s C waves. Chen *et al*. postulated that unspecified low frequency oscillations may modulate CSF motion^4^. Signatures of much lower frequency oscillations in SAS width were observed by Frydrychowski *et al*.^17^ during the development of a novel method for the measurement of SAS width known as near-infrared transillumination-backscattering sounding (NIR-T/BSS) but were not investigated further.

NIR-T/BSS uses infrared light as a radiation source^17^. The principle of NIR-T/BSS is built on the assumption that by proper manipulation of the distance between the source and detectors, the infrared light absorption of haemoglobin can be removed and the SAS can be used as an optical duct^17^. Changes in the SAS width translate into changes in the volume of this duct, thus directly affecting the amount of radiation reaching the detector. To ensure that the signal observed is truly related to the SAS width, extracranial contamination is removed using one detector placed close to the source (the proximal detector), and another detector placed further away (the distal detector)^18^. Although the theoretical background of the NIR-T/BSS technique has been established for some time, more recently it has been verified as a reliable technique for the assessment of SAS width through extensive development. It has since been shown that SAS width changes measured with magnetic resonance imaging and NIRT-B/SS demonstrate high interdependence between the methods (r = 0.81, p < 0.001)^19^. Further validations of the NIR-T/BSS system have demonstrated SAS width oscillations at the cardiac and respiratory frequencies^20^. SAS width signals have recently been collected from elite apnoea divers^21^, although external reproduction of the method remains limited. The theoretical and practical foundations of the NIR-T/BSS method were described in more detail in earlier studies^22^, and are briefly discussed in the Methods section.

The properties of biological oscillations vary in time due to naturally occurring physiological perturbations. The wavelet transform is a time-frequency analysis method that provides optimal resolution for high and low frequencies and gives information about any changes that occur in time^10,23^. The method allows full characterisation of the underlying dynamics of an oscillatory biological system over time, with no prior assumptions, making it ideal for the analysis of biological systems in their resting state^24^. As these oscillations are expected to have very low frequencies, they need to be recorded at rest, with no artificial perturbations, over a long period of time in order to investigate the naturally present dynamics^24^.

In this study we provide the first description of SAS width oscillations in a wide frequency range from 0.005 to 2 Hz, in the resting state, by combining NIR-T/BSS and wavelet analysis. This frequency range encompasses the known frequencies of blood flow oscillations discussed above, and allows the investigation of the hypothesis that low frequency oscillations are observable in SAS width. The relationship between blood pressure (BP) and SAS width oscillations was also investigated. CSF circulation is increasingly recognised as a critical factor in white matter disruption in various pathophysiological states such as multiple sclerosis and hypertension. We propose a new method for the analysis of CSF fluctuations noninvasively, and in the resting state, which may provide new opportunities for research and diagnostics and increase understanding of brain homeostasis.

## Results

**Figure 1:**
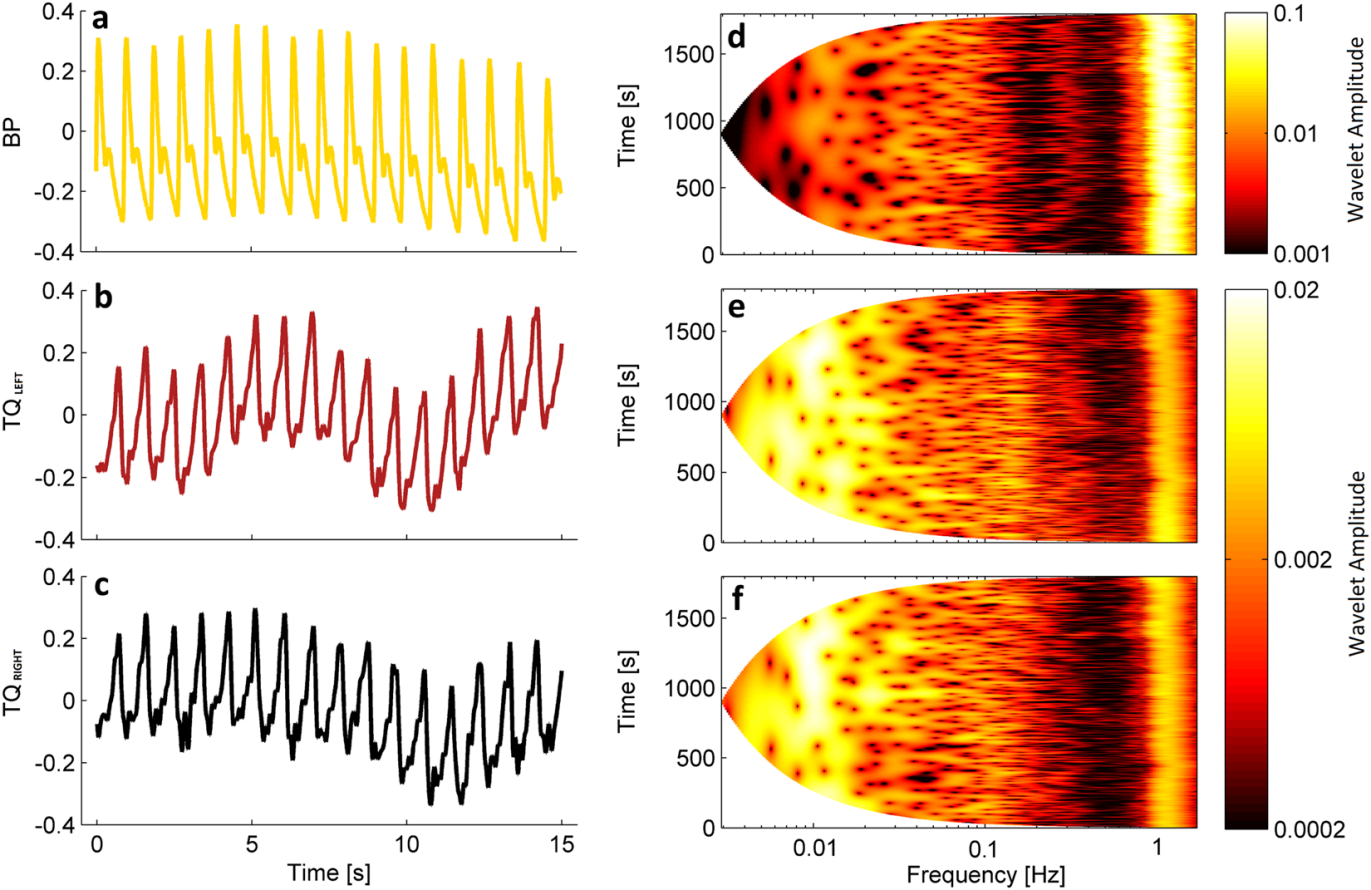
(**a**–**c**) Simultaneous segments (15 second) recordings of (**a**) blood pressure, (**b**) SAS width (left hemisphere) and (**c**) SAS width (right hemisphere) signals measured for one subject. (**d**–**f**) Wavelet transforms of the whole recording (30 minutes) for each signal shown.

Three signals were recorded simultaneously from 36 subjects: blood pressure (BP), TQ_LEFT_ (SAS width in left hemisphere) and TQ_RIGHT_ (SAS width in right hemisphere). Subject characteristics are shown in Table 1. Example signal segments are shown in Fig. 1(a–c).

**Table 1:** Subject characteristics.

	Males (n = 22)	Females (n = 14)
Age (years)	27.02 ± 8.41	24.53 ± 6.19
BMI (kg/m^2^)	25.21 ± 3.37	23.81 ± 3.32
TQ_LEFT_ (AU)	1085.32 ± 83.21	1412.14 ± 91.16
TQ_RIGHT_ (AU)	1242.47 ± 81.56	1373.73 ± 91.82
SBP (mmHg)	121.53 ± 5.41	119.36 ± 7.23
DBP (mmHg)	71.86 ± 6.82	70.23 ± 4.42
HR (beats/min)	61.74 ± 7.43	69.21 ± 6.75
RR (breathes/min)	16.23 ± 3.13	16.98 ± 3.03
SaO_2_ (%)	97.86 ± 1.15	98.12 ± 1.02

Values shown are mean ± standard deviation.

**Figure 2:**
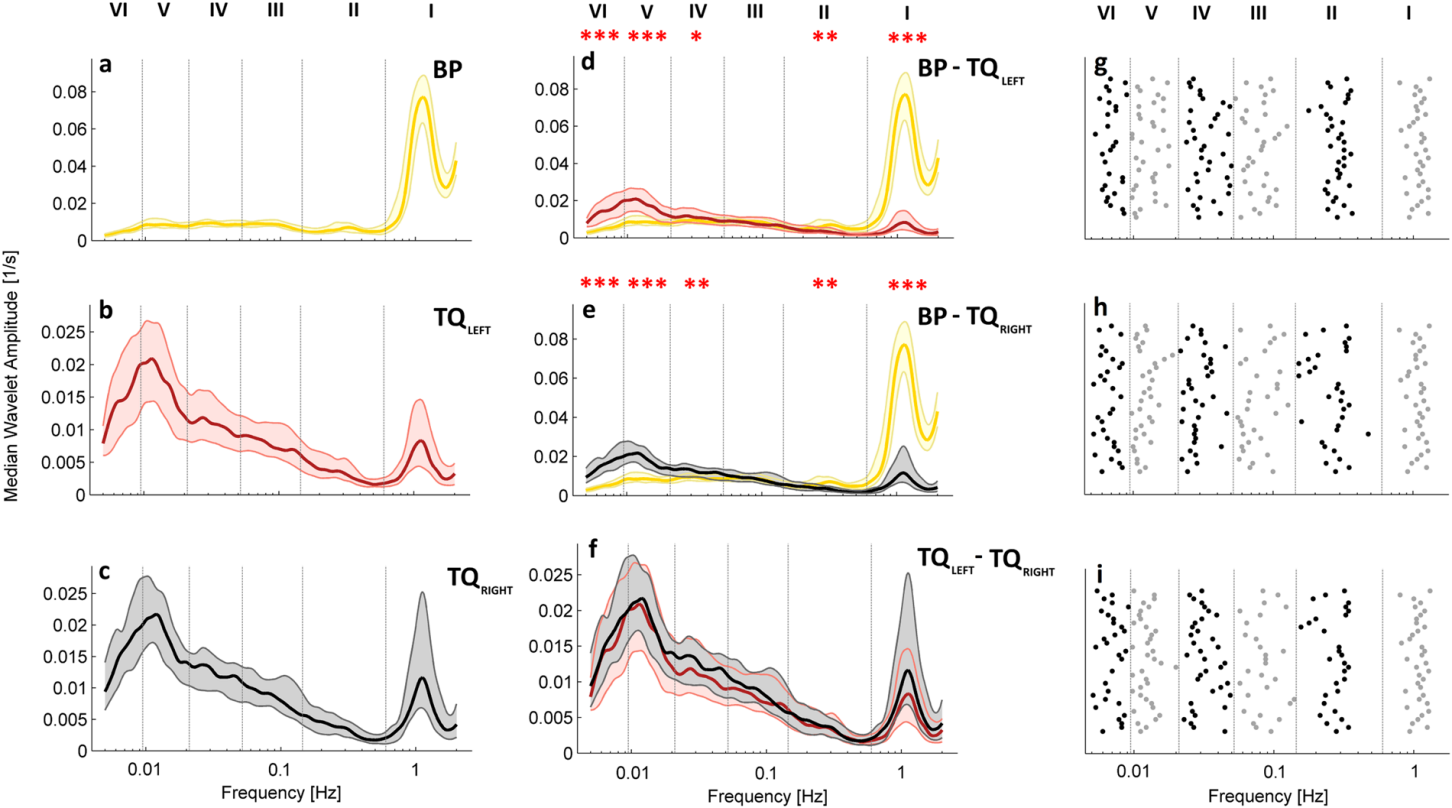
(**a**–**c**) Median (thick lines) of the time-averaged wavelet transforms of signals recorded in all 36 subjects: BP (**a**) and SAS (**b**—left hemisphere, **c**—right hemisphere) obtained from 30 minute recordings. (**d**–**f**) Median (thick lines) of the time-averaged wavelet transforms of (**d**) BP and SAS—left hemisphere, (**e**) BP and SAS—right hemisphere, and (**f**) SAS—left and right hemispheres, all obtained from 30 minute recordings. Shaded areas indicate the inter-quartile range (25^th^, 75^th^ percentiles). *p < 0.05; **p < 0.01; ***p < 0.001. (**g**–**i**) The position of peaks in the time-averaged wavelet transforms of the (**g**) BP and (**h**,**i**) the SAS width in the left and right hemispheres, respectively. The y-displacement of each point corresponds to a particular subject number organized in the same order for all three signals.

To assess the spectral content of these signals, their continuous wavelet transforms were calculated, as shown in Fig. 1(d–f), and their time averages compared between signal types (see Fig. 2). Cardiac oscillations with frequencies around 1 Hz are clearly visible for the whole duration of all signals, but there are also clear oscillations at much lower frequencies (below 0.03 Hz). The latter are much more prominent in the SAS signals recorded in both hemispheres than in the BP signals. The spectra were separated into the frequency intervals described above, and the power compared between signal types. The most significant differences (*p* < 0.001) were observed between BP and SAS spectra in the frequency intervals associated with cardiac (I) and endothelial (V& VI) activity. No significant differences were observed between the spectra of the left and right SAS signals in any frequency interval.

As observed in the dynamics of blood flow^10^, clear peaks were present in the spectra of the SAS width signals. The frequencies of these peaks are plotted in Fig. 2(g–i). The locations of these peaks result in clear bands in the SAS spectra, thus justifying the use of the six previously discussed frequency bands to describe the SAS signals.

To characterise the shape of the spectra in SAS signals, the significance of the difference in mean amplitude between intervals was calculated between the cardiac and respiratory intervals (I & II) and the endothelial intervals (V & VI). Values are shown in Table 2. The results demonstrate that the endothelial spectral amplitude is significantly higher in SAS signals than the cardiac and respiratory amplitude. This result is in contrast to the BP spectra, where the dominant component is the cardiac activity.

**Table 2:** p-values for the differences between wavelet transform mean amplitude in frequency intervals for SAS signals.

	TQ_LEFT_	TQ_RIGHT_
I vs. V	7.81 ∙ 10^−12^	2.31 ∙ 10^−9^
I vs. VI	1.31 ∙ 10^−11^	4.56 ∙ 10^−8^
II vs. V	1.45 ∙ 10^−12^	2.02 ∙ 10^−12^
II vs. VI	1.84 ∙ 10^−11^	6.68 ∙ 10^−12^

### Phase coherence and phase difference

**Figure 3:**
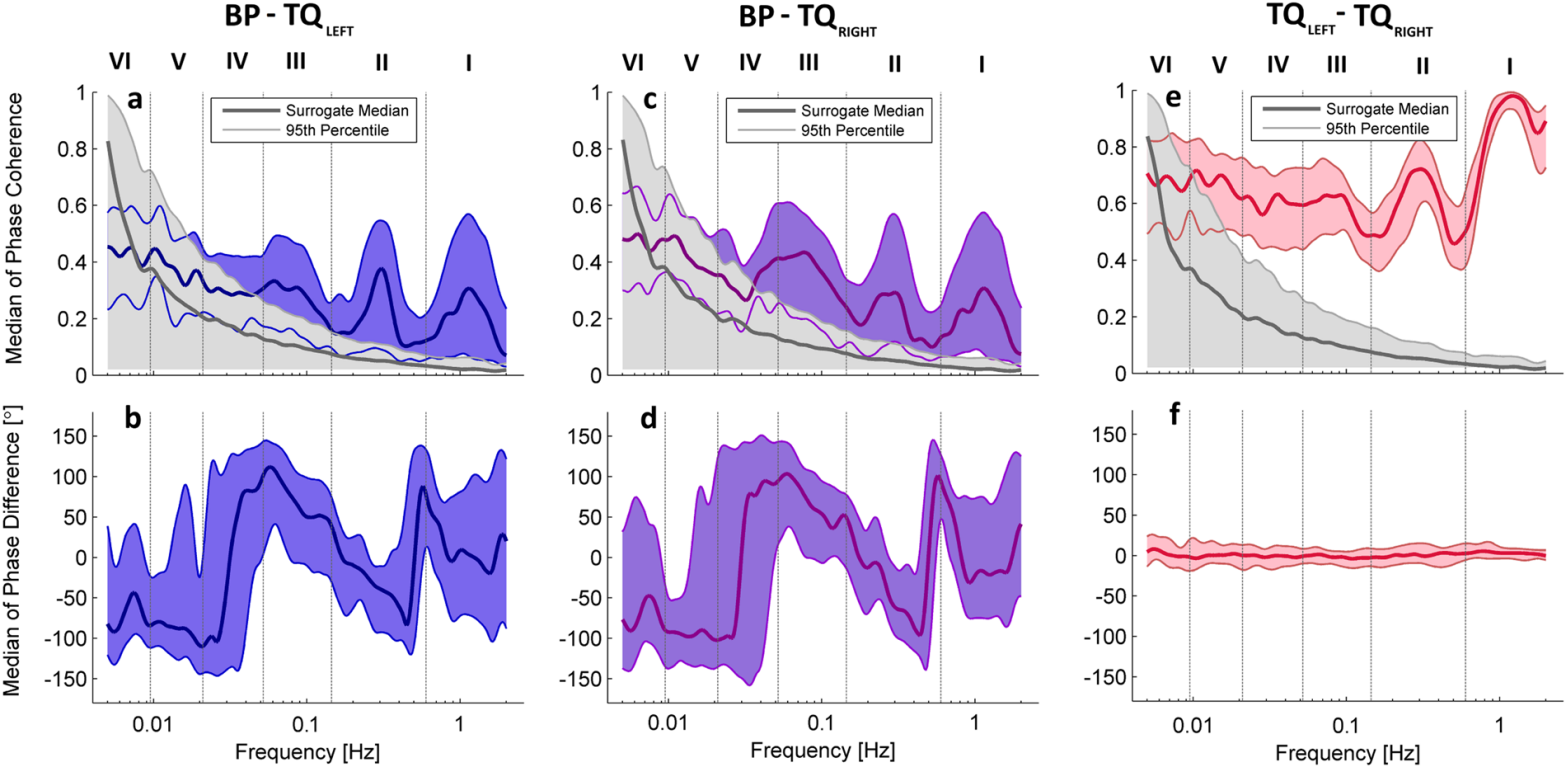
Median (thick lines) of wavelet phase coherence between (**a**) BP and SAS (left hemisphere), (**b**) BP and SAS (right hemisphere), and (**c**) SAS right hemisphere vs SAS left hemisphere. (**b**,**d**,**f**) Phase differences for the coherence in (**a**,**c**,**e**). Coloured shading indicates the interquartile range for 36 subjects. Coherence below the 95^th^ percentile of the surrogates (light grey line and shading) is not considered significant.

Wavelet phase coherence, and the corresponding phase difference (see Methods), were calculated between BP and both (left and right) SAS signals, and directly between the SAS signals (see Fig. 3). Phase coherence at each frequency was considered significant if its value was above the 95^th^ percentile of 1260 (2-permutations of 36 subjects) intersubject surrogates. Phase difference was only taken into consideration at the frequencies where significant coherence was observed. Significant coherence was found in the cardiac, respiration and myogenic frequency intervals when comparing BP and SAS width signals from both hemispheres. Statistically significant coherence between SAS signals from both hemispheres was also obtained at much lower frequencies, down to the NO-dependent endothelial interval. The positive (negative) value of the phase difference for BP and SAS means that the phase of the SAS (BP) signal is leading. For the significant phase coherence (I, II and III frequency interval) the value of phase difference varies at different frequencies. At the cardiac and respiration frequencies, the value of the phase difference is close to zero and we do not observe large phase lag between the two signals. For the whole myogenic frequency interval the phase difference is positive which means that the SAS signal is leading. For the phase coherence between SAS signals the positive (negative) value of phase difference means that the TQ_LEFT_ (TQ_RIGHT_) signal is leading. The phase difference is close to zero for the whole statistically significant frequency interval in this case, showing that there is no phase lag between SAS signals. Correlation analysis was also used to search for relationships between the wavelet phases coherence between SAS and BP signals and age. Significant correlations with age were not observed.

**Figure 4:**
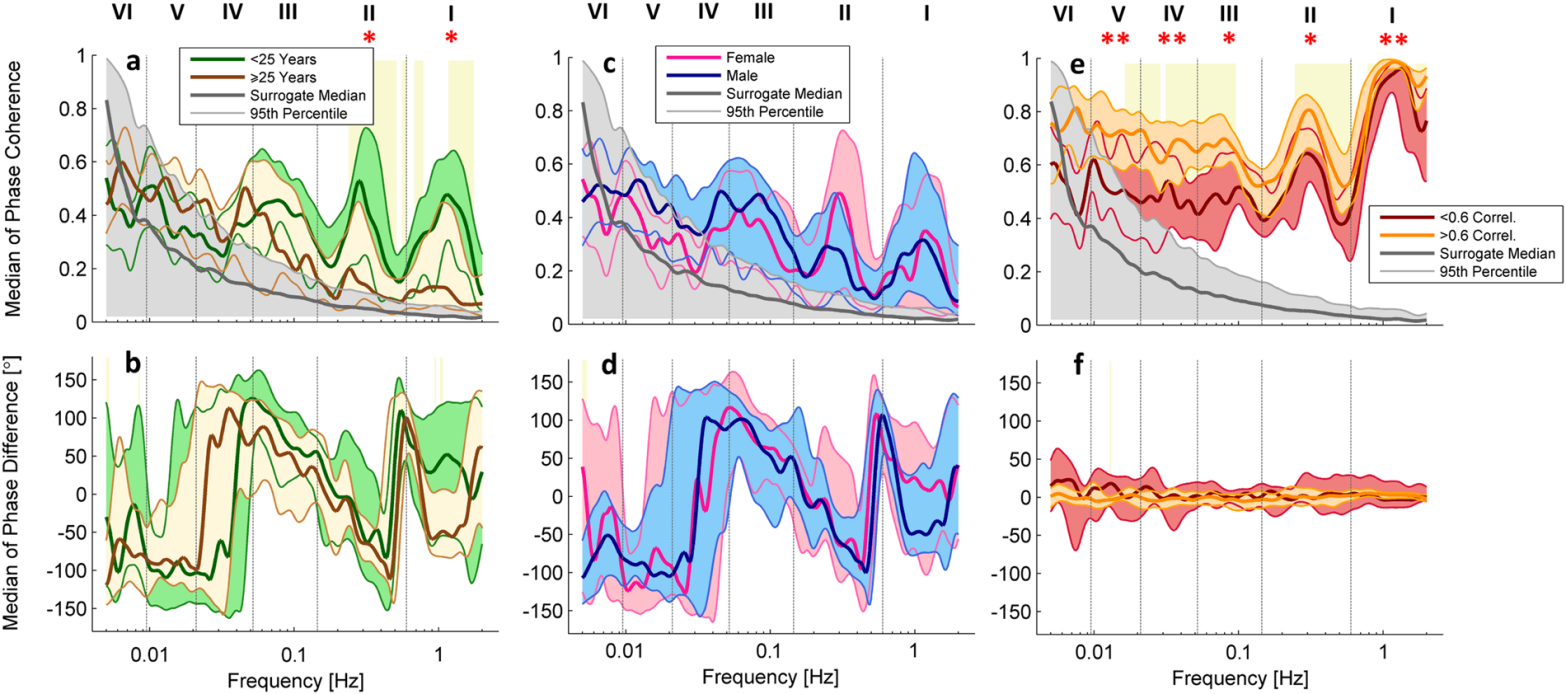
Median (thick lines) of wavelet phase coherence between (**a**) Subjects above and below 25 years of age, (**b**) male and female subjects, and (**c**) subjects with high or low correlation between SAS signals. (**b**,**d**,**f**) Phase differences for the coherence in (**a**,**c**,**e**). Coloured shading indicates the interquartile range for 36 subjects. Coherence below the 95^th^ percentile of the surrogates (light grey line and shading) is not considered significant.

The coherence results were investigated to ascertain whether gender or age differences affect these results. A third parameter was also investigated, the relationship between the correlation of SAS signals and the observed wavelet phase coherence between the signals. These comparisons are presented in Fig. 4. Significant differences were found in the coherence between the different age groups, consisting of subjects below and above or equal to the age of 25, but not between genders. The difference in coherence with age was found in the frequency intervals associated with respiratory and cardiac activity. As expected, subjects that showed significantly higher correlation between the two SAS signals also showed significantly higher phase coherence between the same signals (Fig. 4e). Significant differences were observed in all frequency intervals except the lowest endothelial interval (VI), which is likely due to the effects of the surrogates and the bias of phase coherence at low frequencies.

## Discussion

We investigated the dynamics of sub-arachnoid space (SAS) width oscillations, and how they are related to the blood pressure and, indirectly, blood flow. The main finding of the study were the prominent oscillations observed at low frequencies in the TQ_LEFT_ and TQ_RIGHT_ SAS width signals across a wide frequency range (0.005–2 Hz) (see Fig. 1e,f). Further investigation into these oscillations showed that, in contrast to the blood pressure (BP) signal, the amplitude of the lowest frequency oscillations, those associated with endothelial activity, is much higher than the amplitude of the cardiac and respiratory oscillations in the same signals (see Table 2). Analysis of the locations of the peaks in the wavelet transform showed similarities with the widely accepted frequency intervals that are used to characterise blood flow oscillations, providing evidence that low frequency SAS oscillations may have similar origins.

We also investigated how these oscillations are related to the blood pressure using wavelet phase coherence (see Fig. 3a,c). Significant coherence was found between BP signals recorded from the finger and SAS width signals recorded from both sides of the head (see Fig. 3a,c). Clear coherence peaks were observed in the cardiac, respiration and myogenic intervals (see Fig. 3a,c). Phase difference analysis suggests that at the cardiac and respiratory frequencies both signals are independent and oscillations are generated centrally by the heart and lungs, respectively^23^. On the contrary, the leading of the SAS phase in the myogenic frequency may indicate active local processes adjusting vessel activity to the metabolic requirements of the brain^25^. Significant phase coherence was also observed between the two SAS signals in the same intervals, and also in the lower neurogenic and endothelial frequency intervals (see Fig. 3e).

No significant differences due to gender were observed in the wavelet transforms or in the phase coherence analysis (see Fig. 4c), however age-dependent differences in the wavelet phase coherence between BP and SAS signals were demonstrated (see Fig. 4a). These are very interesting findings, especially considering the relative youth of the subjects included in this study, and may suggest that cerebrovascular ageing begins earlier than expected. Several authors have demonstrated that cerebral microcirculation is impaired with age, which is reflected in declining low frequency oscillations (0.005–0.145 Hz)^26–28^. As the number of individuals investigated was relatively small and the age range (18–42 years) does not cover the critical span of life where ageing affects health, this finding should be confirmed in a larger cohort with a wider age range. Nevertheless, it demonstrates the potential of the proposed combination of NIR-T/BSS and wavelet analysis methods for the investigation of CSF dynamics.

Abnormal CSF pulsatility has been implicated in subjects with impaired jugular outflow^29–32^ and in patients suffering from diseases such as multiple sclerosis or hypertension^29,32,33^. However, all of these studies analysed CSF pulsatility at the cardiac frequency. Heart and respiratory driven CSF fluctuations are largely of a mechanical nature. CSF is proposed to be part of a Windkessel mechanism, absorbing abrupt systolic blood inflow, smoothing the capillary blood flow, and facilitating diastolic jugular outflow^32^. These mechanisms become less efficient with ageing and are particularly dysfunctional in patients suffering from dementia^32^. Our results suggest that age-related changes in CSF pulsatility may be detected at a relatively early stage, though this effect needs to be verified in older subjects.

It has been shown that cardiorespiratory coupling is impaired with ageing^34–36^. Consequently, BP respiration modulation diminishes and transmission of BP oscillations to CSF is also less evident. Inspiratory-driven venous return is likely governed by different age-dependent dynamics^34^. This provides a possible explanation for the reduction of phase similarity between BP and SAS width oscillations with age at the respiratory frequency.

Correlation between left and right hemisphere SAS width oscillations was also investigated, and found to vary significantly between subjects. Separating subjects with high or low SAS dynamics correlations into two groups, we identified differences in phase coherence in most frequency intervals. We consider that these differences could have arisen from the activation of the brain noradrenergic system (α_2_-receptors in prefrontal cortex in particular) by the different states of mind of the participants, in whom the synchrony in brain regions could have been affected by thinking, planning, or memory retrieval. It should be noted that there was a slightly higher SAS correlation in the female group (although not statistically significant), which may suggest a trend towards higher interhemispheric connectivity in females, which is in line with existing knowledge^37,38^.

We have proposed a non-invasive method, based on NIR-T/BSS and time-frequency analysis, for the investigation of SAS width dynamics in humans. By combining uniquely the power of oscillations associated with individual physiological processes within the frequency range 0.005–2 Hz and SAS signals recorded with NIR-T/BSS we obtain a method that reveals valuable functional information about the dynamics of the SAS width and its relationship to the BP. Combining NIR-T/BSS with advanced signal analysis tools is a promising approach in describing the interrelations and pathways involved in white matter damage in several brain diseases or cardiovascular and neurological origin. The observed CSF oscillations could explain the occurrence of specific intracranial pressure waves, i.e. the B-waves which can be observed especially in pathological situations like head injury or hydrocephalus. Future studies could provide further insights into various neurodegenerative and ageing related diseases through the investigation of coherences between NIR-T/BSS and NIRS or EEG signals. This could open new frontiers in science and improve diagnostic and follow-up procedures.

## Methods

### Measurements

Thirty-six healthy subjects were recruited to the study. All subjects received detailed information about the study objectives and any potential adverse reactions, and they provided written informed consent to participate in the study. Signals were collected at the University of Regina, Canada and the Medical University of Gdansk, Poland. The experimental protocol and the study were approved by the Research Ethics Committees of the University of Regina (REB 55R1213) and Medical University of Gdansk (NKBBN/572/2014–2015). The study conformed to the standards set by the Declaration of Helsinki. Participants were all non-smokers, did not suffer with any known disorders, and were not taking any medication, as confirmed by a general and neurological health demographic questionnaire. Exclusion criteria included the consumption of any caffeine containing food and beverages for 8 hours prior to the measurements. Participants were also asked to refrain from exercise training for a minimum of 12 hours prior to testing, and from the consumption of alcohol for 24 hours before the test. All tests were conducted in a comfortable quiet room pre-set to a temperature of 18–20 °C with low ambient light. Participants were instructed to lie down on a bed with a pillow to support their head. A blanket was provided if required. The atmosphere was normobaric throughout testing.

Blood pressure (BP) was measured using a Finometer (Finapres Medical Systems, Arnhem, The Netherlands). This system uses a finger-cuff to assess beat-to-beat blood pressure from the left middle finger. Finger blood pressure was initially calibrated against brachial arterial pressure (PhysioCal), but then the calibration was turned off during the measurement to obtain an unaltered waveform^39^.

**Figure 5:**
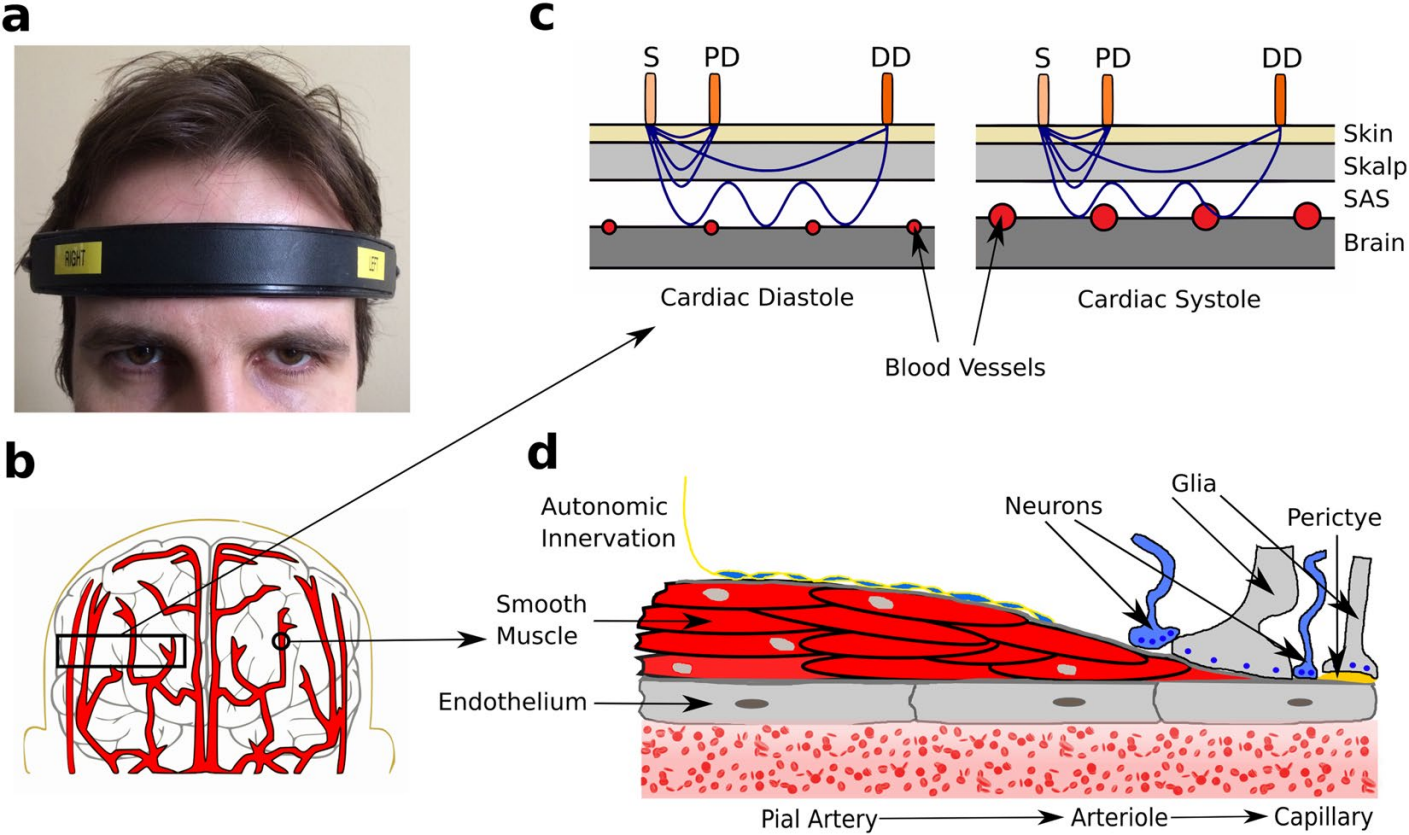
Location of NIR-T/BSS sensors on right and left hemispheres. (**b**) A simplified model of cerebral vessels located in the frontal part of the head. (**c**) A simplified diagram illustrating the influence of heart induced pulsatile changes during diastolic and systolic phases, which directly affect the NIR-T/BSS radiation propagation within the tissues in the head. (**d**) Model of cerebrovascular vessels, from large pial arteries to small capillaries.

The SAS width was recorded separately for right and left hemispheres with two identical head-mounted sensors of the NIR-T/BSS device (SAS 100 monitor, NIRTI SA, Wierzbice, Poland). A single sensor-detector module of NIR-T/BSS (on one side of the head) consists of the source (S) and two photo-detectors (PD—proximal detector and DD—distal detector). The PD and DD were positioned 7 and 28 mm away from the source, respectively. These distances have been shown to be optimal based on Monte Carlo simulations^22^. Figure 5(a) illustrates the symmetrical placement of the NIR-T/BSS headband onto the forehead during the measurements. The near infrared radiation emitted from the source penetrates the skin, the skull and tissue layers, propagates through the SAS, and returns to the detectors, again through tissues, skull and skin (see Fig. 5(c)). Figure 5(c) illustrates the pulsatile modulation of near infrared radiation related to cardiac-induced pulsatile changes of blood vessel volume in the SAS layer. Increased blood volume during the systolic phase results in a decrease in the width of the SAS, and thus a reduction in the amount of radiation propagated from the source to the detector. Figure 5(b) shows a simplified structure of cerebral vessels. Large cerebral arteries arising from the circle of Willis branch out into smaller pial arteries, arterioles, and capillaries. The vessels travel on the surface of the brain, across the subarachnoid space and enter into the substance of the brain. Figure 5(d) shows the vessel structure in more detail.

Relatively short distance between source light and detectors helps to limit extracranial contamination^40,41^. Using the signal from the PD, the absorption from skin and bone is eliminated. The quotient of the remaining signals is sensitive to changes in the width of the SAS, and is known as the transillumination quotient (TQ)^17^. The intensity of infrared radiation at one wavelength is registered by the two sensors that provide information about the attenuation of the original signal in tissues. The infrared radiation used has a wavelength of 880 nm, which has been proven to easily penetrate tissues and importantly, be almost completely insensitive to changes in haemoglobin oxygen saturation^42–45^. To ensure that the NIR-T/BSS method is sensitive only to SAS width variations, Monte Carlo simulations were recently performed^46^, which showed that for the chosen source-detector distances the dominant contribution to the TQ signal is SAS width rather than the absorption of the brain.

Although using a similar radiation source, NIR-T/BSS is distinct from near-infrared spectroscopy (NIRS). NIR-T/BSS uses only one wavelength, while NIRS uses several. The frequency modulation of the source in NIR-T/BSS is much less than in NIRS, and any physiological disturbances are immediately visible in NIR-T/BSS. This is in contrast to NIRS, where physiological changes occur with some delay. The most important limitation of NIR-T/BSS is that TQ is not a measure of the absolute width of the SAS expressed e.g. in millimetres, but provides information about the changes in SAS width over time. This is due to the effects of anatomical differences in scalp and tissue thickness. Monte Carlo simulations have been used to investigate the effects of varying thicknesses of SAS and skull bone^18^. Strong correlations were observed between the power of the reflected stream of photons and the varying bone-brain distance. This conclusion was fully consistent with the findings presented by other authors^47,48^.

Raw BP and SAS width signals were recorded for 30 minutes and imported into PowerLab 16/30 (AD instruments, Colorado Springs, Colorado, USA) and then viewed as live data in LabChart Pro. The signals were digitized with 16-bit resolution at a sampling rate of 300 Hz. Prior to analysis, the BP and SAS signals were detrended using a moving average with a window size of 120 s and normalized by subtraction of their mean and division by their standard deviation. The signals were also downsampled to 10 Hz.

### Analysis

The dynamics in the recorded signals were investigated using the wavelet transform and wavelet phase coherence. The wavelet transform is a time-frequency analysis method that provides the opportunity to observe how the frequency content of a signal changes over time. This makes it ideal for application to biological signals, which are consistently time-varying. Another advantage of the wavelet transform is the logarithmic frequency scale that it provides, allowing a much higher resolution at the low frequencies at which biological oscillations usually manifest^10^. The wavelet transform was previously employed to reveal the periodic components in blood flow signals^10^, and as we expect similar modulations to be present in SAS signals, we consider this the optimal approach.

The wavelet transform is defined as:

1 $$W(s,t)=\frac{1}{\sqrt{s}}{\int }_{-\infty }^{+\infty }\phi (\frac{u-t}{s})g(u)du,$$

where *W(s,t)* is the wavelet coefficient, *g(u)* is the time series, and *φ* is the Morlet mother wavelet, scaled by factor *s* and translated in time by *t*. The Morlet mother wavelet is defined by the equation:

2 $$\phi (u)=\frac{1}{\sqrt[4]{\pi }}\exp (-i2\pi u)\exp (-0.5{u}^{2}),$$

where $i=\sqrt{-1}$. The Morlet wavelet is used due to its good localisation of events in time and frequency due to its Gaussian shape^49,50^. Signals of 30 minute duration with a sampling rate of 100 Hz enable reliable calculation of the frequency spectrum in the range 0.003–50 Hz. Here we focus on the interval between 0.005–2 Hz, where the oscillations described above would be expected to manifest if present. When using the Morlet wavelet transform, the obtained coefficients are complex numbers, *X*(*ω*_*k*_, *t*_*n*_) = *X*_*k,n*_=*a*_*k,n*_ + *ib*_*k,n*_, providing both amplitude ($|{X}_{k,n}|=\sqrt{{a}_{k,n}^{2}+{b}_{k,n}^{2}}$), and phase (${\theta }_{k,n}=\arctan (\frac{{b}_{k,n}}{{a}_{k,n}})$) information for each point in frequency and time. This allows phase information to be studied independently to amplitude modulations. This separation can be exploited in the method of wavelet phase coherence (WPCO), which uses this phase information to determine whether the oscillations detected are significantly correlated over time. To calculate the WPCO, the instantaneous phases at each time and frequency point are extracted for both signals (*θ*_1*k,n*_, *θ*_2*k,n*_). Phase coherence is then^51,52^:

3 $${C}_{\theta }({f}_{k})=\frac{1}{n}|\sum _{t=1}^{n}\exp [i({\theta }_{2k,n}-{\theta }_{1k,n})]|.$$

The value of the WPCO function *C*_*θ*_(*f*_*k*_) will be between 0 and 1. The phase difference of two unrelated oscillations will continuously change with time, giving a phase coherence that tends to zero. If the oscillations are related and their phase difference remains almost constant, the value of the phase coherence will tend to 1.

Wavelet phase coherence examines phase relationships only, and is not enhanced by any amplitude relationships that may also be present in the signals. In our previous studies we have shown that BP-SAS amplitude similarity at the cardiac frequency is affected by several stimuli such as apnoea^20^ or hypoxia^53^. In these cases the wavelet coherence approach which also takes amplitude into account may be more appropriate. However, in the resting state presented in this study, wavelet phase coherence is sufficient.

Additionally, we can calculate the phase difference Δ*θ*_*k*_ between two signals according to

4 $${\rm{\Delta }}{\theta }_{k}=arctan(\frac{\frac{1}{n}{\sum }_{t=1}^{n}sin({\theta }_{2k,n}-{\theta }_{1k,n})}{\frac{1}{n}{\sum }_{t=1}^{n}cos({\theta }_{2k,n}-{\theta }_{1k,n})}).$$

The value of Δ*θ*_*k*_ is between −180° and 180° and provides information about the phase lag of one oscillator compared to the other.

Within a time-frequency representation of a signal, there are naturally less cycles of oscillations the lower in frequency that we consider. This can cause artificially increased wavelet phase coherence at low frequencies. This bias has been demonstrated using pairs of unrelated white noise data, for which the wavelet phase coherence was shown to increase at low frequencies^51,54^. Therefore, to obtain a reliable coherence value, surrogate data testing should be used. Surrogate data testing is a method that provides a ‘statistical zero’, or the expected range of values of a discriminating statistic in data which is the same as the data to be tested, but is missing the property to be tested. In this case, we wish to calculate the expected values of wavelet phase coherence in data where there is definitely no coherence. If the coherence calculated in the real data is higher than the threshold set by the range of surrogate values, it can be considered as significant, with a confidence dependent on the threshold used. Many different surrogate types have been used. In this work we used intersubject surrogates^35,55^, which rely on the assumption that similar mismatched signals recorded from different subjects will not be coherent. This method was shown to provide similar results to the widely used iterative amplitude adjusted Fourier transform (IAAFT) surrogates^56,57^ for the calculation of wavelet phase coherence^35^.

Nonparametric statistical tests were used for all comparisons, to avoid the assumption of normality in the results. The Wilcoxon rank sum test was used to compare whether the median of different groups was significantly different, both for comparisons of wavelet amplitude and wavelet phase coherence.

We examined the effects of three parameters on our results: age, sex and SAS correlation. For each parameter, the subject population were split into two groups. For each parameter comparison, it was ensured that there were no significant differences in the other parameters, for example, there were no significant age differences in the two subject groups when performing statistical tests for sex differences. In cases where significant differences were found between groups, for example the difference in BP vs. SAS width coherence in subjects below and above or equal to the age of 25, the effect size was evaluated using the statistical ‘pwr’ package in the R statistical programming language. Significant differences were only reported if the effect size exceeded the threshold required to ensure a test power of at least 0.8, i.e. β = 0.2.
